# Effect of bilateral inferior oblique partial myectomy on V pattern exotropia with inferior oblique overaction

**DOI:** 10.1186/s12886-022-02456-1

**Published:** 2022-05-21

**Authors:** Tianke Yang, Chunwen Chen, Wenxiu Ma, Yubing Duan, Qin Zhu, Jingyan Yao

**Affiliations:** 1 Department of Ophthalmology, The First Affiliated Hospital of Soochow University, Suzhou, 215006 Jiangsu China; 2 Eye Institute and Department of Ophthalmology, Eye & ENT Hospital, Fudan University, Shanghai, 200031 China; 3 Department of Ophthalmology, University Hospital, LMU Munich, Mathildenstrasse 8, 80336 Munich, Germany

**Keywords:** V-pattern exotropia, Inferior oblique overaction, Inferior oblique partial myectomy, Fovea-disc angle

## Abstract

**Purpose:**

To compare the effect of bilateral inferior oblique partial myectomy on V-pattern exotropia patients with bilateral symmetric inferior oblique overaction (IOOA) and asymmetric IOOA.

**Methods:**

This was a retrospective study including 53 V-pattern exotropia patients with bilateral IOOA of all grades who underwent bilateral inferior oblique partial myectomy. Success was defined as the elimination of the IOOA and the collapse of the V pattern at the final follow-up. The fovea-disc angle (FDA) and V-pattern exotropia were compared before and after surgery.

**Results:**

This study included 53 V-pattern exotropia patients, containing 29 patients with symmetric IOOA (Group I) and 24 patients with asymmetric IOOA (Group II). The last follow-up ranged from 3 to 16 months (mean of 5 months). After myectomy, 3 eyes in Group I and 2 eyes in Group II were observed with residual grade 1 IOOA. The surgical success rates of IOOA correction in Group I and Group II were 96% and 95%, respectively. The difference was not statistically significant (*P* = 0.808). V-pattern exotropia collapsed with residual 2 (min. 0, max. 6) PD for Group I and 2 (min. 0, max. 10) PD for Group II, and there was a statistically significant difference between pre- and postoperative V-pattern exotropia in the two groups (*P* = 0.000). No inferior oblique (IO) underaction or antielevation syndrome (AES) was found in either group. The average preoperative FDA of the right eye and the left eye was (8.93 ± 4.34)° and (10.86 ± 4.27)° in Group I and (9.08 ± 4.92)° and (11.00 ± 5.69)° in Group II. There was a significant difference in preoperative FDA between the right eye and the left eye in the two groups (Group I *p* = 0.029; Group II *p* = 0.038).

**Conclusions:**

Bilateral inferior oblique partial myectomy can bring “symmetric” effectiveness in the correction of IOOA and FDA. It can potentially be used as a safe and successful treatment for V-pattern exotropia with bilateral IOOA. In addition, the FDA may be a promising index for evaluating fundus extorsion.

## Introduction

A- and V-pattern strabismus are unique forms of vertical incomitant strabismus characterized by a change in the horizontal deviation from the primary position to the upgaze and downgaze [1]. The A pattern generally indicates a difference in exodeviation between the upgaze and downgaze of greater than 10 prism diopters (PD), and the V pattern indicates a minimum difference of 15 PD [2]. Although the precise etiology and pathogenesis remain unclear, oblique muscle dysfunction is accepted as a predominant mechanism for the A and V patterns [3–7]. The oblique muscle dysfunction etiology indicates that A-pattern strabismus is frequently associated with superior oblique overaction (SOOA), and V-pattern strabismus is closely related to inferior oblique overaction (IOOA).

IOOA in V-pattern strabismus can be divided into two main categories: primary IOOA and secondary IOOA. In the clinic, inferior oblique (IO) weakening procedures are mainly used to treat primary or secondary IOOA and associated V pattern strabismus. For the surgical treatment of IOOA, several different techniques have been developed. Costenbader FD et al. analyzed three different IO weakening procedures and suggested that the effects of myotomy at its origin, recession at the scleral insertion and partial myectomy were similar [8]. Ercan Ozsoy et al. studied the efficacy of IO weakening treatment in 179 patients with IOOA and showed that the cure rates of IO recession, myectomy and anterior transposition were 96%, 98.4% and 93.9%, respectively [9]. Antielevation syndrome was found in patients with IOOA who underwent IO anterior transposition.

Several studies have focused on the effects of unilateral IO weakening surgeries on bilateral IOOA [10–12]. The authors found that if only the side of the IO with more severe overaction was performed, IOOA may be aggravated in the fellow eye in 36.1% of patients. Ahmed Awadein et al. studied the effects of bilateral symmetric IO myectomy on patients with asymmetric IOOA [10]. They concluded that IOOA was significantly improved after symmetrical surgery without dysfunctional IO. However, currently reported data examining the effectiveness and safety of symmetric IO partial myectomy for V-pattern exotropia with bilateral IOOA are limited [9–13]. In addition, the efficacy of symmetric IO partial myectomy has not been assessed quantitatively using methodologically sound research. The purpose of this study was to evaluate the efficacy of symmetrical IO partial myectomy for the treatment of V-pattern exotropia with bilateral IOOA and to compare the effectiveness in the correction of symmetric and asymmetric IOOA.

### Patients and methods

This retrospective study was conducted in the First Affiliated Hospital of Soochow University between August 2017 and August 2019. The study was reviewed and approved by the Ethics Committee of the First Affiliated Hospital of Soochow University and complied with the principles of the Declaration of Helsinki. Informed consent from a parent or legal guardian for study participation was obtained for all children. The medical records of V-pattern exotropia patients with bilateral primary or secondary IOOA, either symmetric or asymmetric IOOA, who underwent bilateral symmetric IO partial myectomy were collected. The exclusion criteria for this study are listed below: (1) patients with dissociated vertical deviation, amblyopia, anisometropia, insertion anomalies of IO muscles and ocular structural abnormalities; (2) patients with an extraocular muscle surgery history; and (3) patients with central nervous system abnormalities or other systemic disorders.

Complete ocular examinations, including visual acuity testing, intraocular pressure, cycloplegic refraction, corneal topography, slit-lamp and fundus examinations, were performed on all patients by the same ophthalmologist. The angle of deviation was measured by the alternate prism cover test under optimal corrected visual acuity at both 6 m and 33 cm in the primary position, 25 degrees upgaze and 35 degrees downgaze, respectively [14]. To guarantee accurate data, patients were patched into either eye for more than 30 minutes prior to deviation angle examination, and the examination was performed at least 3 times. For patients with exotropia, the maximum deviation angle was recorded. The Titmus stereotest (Stereo Fly Test, Stereo Optical Company, Inc., Chicago, IL, USA) was performed at a distance of 40 cm. The Titmus stereotest detected stereoacuity varying from 40, 50, 60, 80, 100, 140, 200, 400 to 800 seconds of arc by the circle figures.

Ductions and versions were evaluated in all patients. The grades of IOOA were classified as follows: 1) If the abducting eye was fixating straight in abduction, a slight upshoot of the adducting eye was recorded as grade 1 overaction. 2) Grade 2 overaction was recorded if there was an obvious upshoot of the adducting eye when the abducting eye was in horizontal abduction. 3) A severe upshoot of the adducting eye was considered grade 3 overaction. 4) An extremely severe upshoot of the adducting eye was regarded as grade 4 overaction [15].

**Fig. 1:**
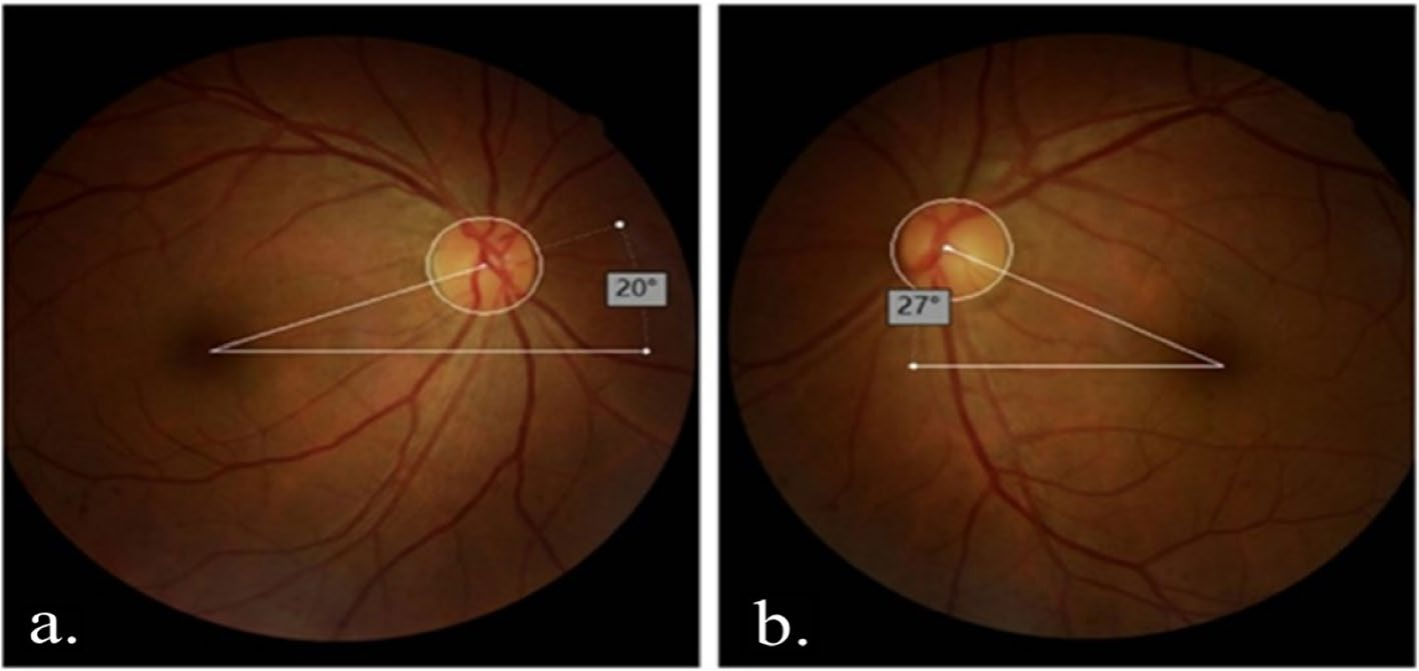
Marks and measurement of the fovea-disc angle with Auto CAD software

All of the patients underwent fundus photography provided by a stereoscopic fundus camera pre- and postoperatively. The patient was asked to keep the head straight and look at an internal fixation with either eye occluded. The fovea-disc angle (FDA) was examined between a horizontal line drawn through the fovea and a line connecting the fovea to the optic disc center from the good focus photograph using Auto CAD 2014 imaging software [16]. The torsion angle was defined as “+” if the fovea was located below the optic disc center, which means extorsion. Conversely, the fovea was located upon the optic disc center, defined as “-” , which means intorsion [17] (Fig. 1).

Residual IOOA, collapse of the V pattern, and FDA were evaluated on postoperative day 1, day 7 and at 1, 3, 6, 12 and 24 months postoperatively. Data recorded at the final follow-up postoperatively were included in this study. The difference in correction between the FDA and IOOA was compared in each group. The successful outcome was regarded as no or a small deviation in primary position (≤ 10 pd), IO muscle function of grade 0 and the collapse of the V pattern.

### Surgical procedure

**Fig. 2:**
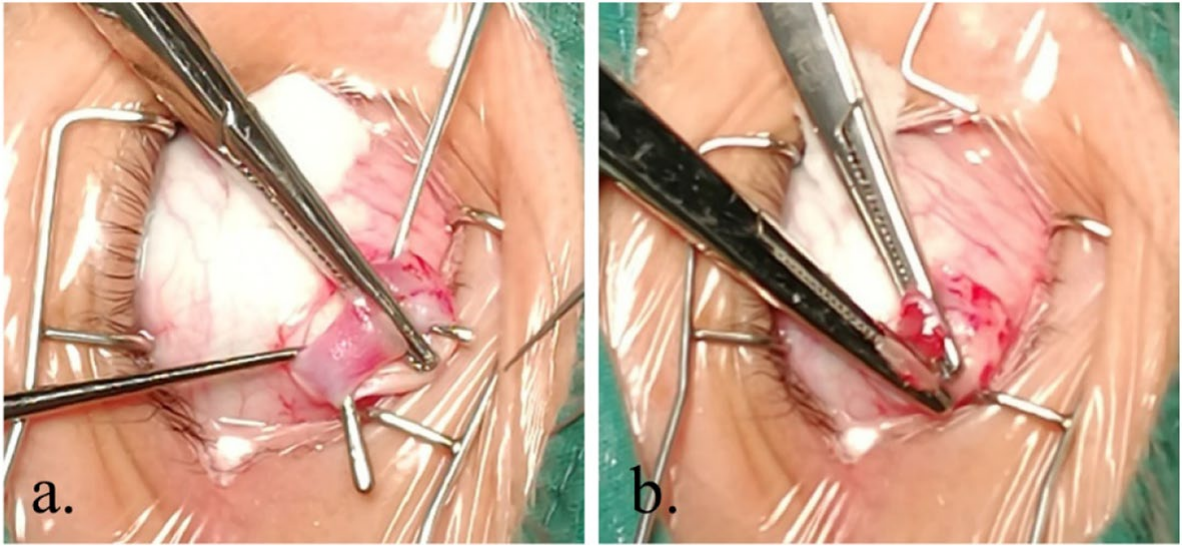
Steps of inferior oblique partial myectomy. **a** Isolating inferior oblique muscle and clamping with small hemostats, **b** Cutting inferior oblique muscle at the temporal hemostatic forceps and excising the segment of inferior oblique muscle from the nasal hemostatic forceps

All procedures were performed under general anesthesia to guarantee the safety of the operation and alleviate patients pain. In all procedures, initially, an 8 mm long standard inferotemporal fornix conjunctival incision parallel to the corneal limbus was made between the inferior rectus and lateral rectus. After Tenon’s capsule was dissected and lifted, operation of the horizontal muscles was performed. The IO was identified and isolated under direct vision using a muscle hook. Then, the surrounding fascia tissue was separated. The IO was stretched and exposed using two muscle hooks. A 3–5 mm segment of muscle from both sides was clamped with two small hemostats. Next, the IO was cut at the temporal hemostatic forceps, and the 3–5 mm segment of the IO muscle was excised from the nasal hemostatic forceps. The insertion site was burned to stop the bleeding. The muscle was released into the tenon’s capsule. Finally, the surgeon closed the conjunctival incisions with 8–0 absorbable sutures and anti-inflammatory ointments (Fig. 2).

### Statistical analysis

All statistical analyses were performed using SPSS software version 25.0 (SPSS Inc. Chicago, IL, USA). Data are presented as the median, minimum (min), and maximum (max) or as percentages. Significance was tested using t tests, Mann–Whitney tests, chi-square tests, and the nonparametric Wilcoxon signed rank test as appropriate. A *P* value under 0.05 was considered significant.

## Results

Overall, this study included 53 V-pattern exotropia patients, containing 29 patients with symmetric IOOA (Group I) and 24 patients with asymmetric IOOA (Group II). Group I included 58 eyes of 29 patients, and Group II included 48 eyes of 24 patients. The median age at surgery was 11 (min. 8, max. 38) years for Group I and 13.5 (min. 9, max. 52) years for Group II. The last follow-up ranged from 3 to 16 months (mean of 5 months). One patient in Group I received bilateral IO partial myectomy, while the other 52 patients underwent IO myectomy combined with horizontal muscle surgery. No significant differences were found between the two groups concerning demographics (Table 1).

**Table 1:** Demographics and surgical approach of the studied groups

	Group I (*n* = 29 patients, 58 eyes)	Group II (*n* = 24 patients, 48 eyes)	*P*
Age (years)	11	13.5	0.122^a^
Median (minimum,maxmum)	(8 ~ 38)	(9 ~ 52)	
Gender (no., %)			0.707^b^
Male	16 (55%)	12 (50%)	
Female	13 (45%)	12 (50%)	
Mean angle of horizontal exotropia (PD)			
33 cm	55.517 ± 28.807 (18 ~ 105)	55.500 ± 24.686 (25 ~ 100)	0.998^c^
6 m	49.414 ± 28.696 (18 ~ 105)	53.500 ± 24.456 (25 ~ 98)	0.584^c^
Surgery approach (no., %)			1.000^d^
IO surgery	1 (3%)	–	
MR/LR + IO surgery	28 (97%)	24 (100%)	
	R-R (*n* = 16)	R-R (*n* = 15)	
	BLRec (*n* = 4)	BLRec (*n* = 3)	
	BLRec + UMRes (*n* = 8)	BLRec + UMRes (*n* = 6)	

*BLRec* Bilateral lateral rectus recession, *UMRes* Unilateral medial rectus resection, *R-R* Unilateral recession and resection. ^a^ Mann–Whitney test; ^b^ Chi-square test; ^c^ t test; ^d^ Fisher’s exact test

The preoperative grades of IOOA in each group were as follows: 32 eyes with grade 2 IOOA (Group I), 26 eyes with grade 3 IOOA (Group I), 10 eyes with grade 1 IOOA (Group II), 22 eyes with grade 2 IOOA (Group II) and 16 eyes with grade 3 IOOA (Group II). At the final follow-up recording postoperatively, 3 eyes with residual grade 1 IOOA (Group I) and 2 eyes with residual grade 1 IOOA (Group II) were observed. The surgical success rates of the asymmetric IOOA group and the symmetric IOOA group were 96% and 95%, respectively. All patients showed no IO underaction. In addition, no patients exhibited AES. Improvement in IOOA and horizontal deviation was illustrated in Tables 2 and 3.

**Table 2:** Change in inferior oblique overaction in the studied groups

	Group I (*n* = 29 patients, 58 eyes)	Group II (*n* = 24 patients, 48 eyes)	*P*
Surgery success (IOOA<grade1) (no., %)	55 (95%)	46 (96%)	0.808^a^
Residual IOOA (no., %)	3 (5%)	2 (4%)	
Grade 1	0/0 (0%)	0/10 (0%)	
Grade 2	2/32 (6%)	0/22 (0%)	
Grade 3	1/26 (94%)	2/16 (1%)	
*P*^*a*^	0.000^*^	0.000^*^	

*P significant; ^a^ Chi-square test

**Table 3:** Change in horizontal deviation and the V pattern in the studied groups

	Group I (*n* = 29 patients, 58 eyes)	Group II (*n* = 24 patients, 48 eyes)	*P*
Corrected horizontal deviation (PD)			
33 cm	51.172 ± 28.853	51.417 ± 26.708	0.975^a^
6 m	45.103 ± 28.287	47.750 ± 26.982	0.731^a^
Preoperative V pattern (PD)	20	20	0.588^b^
Median (minimum, maxmum)	(15 ~ 45)	(15 ~ 63)	
Postoperative V pattern (PD)	2	2	0.210^c^
Median (minimum, maxmum)	(0 ~ 6)	(0 ~ 10)	
*P*^*b*^	0.000^*^	0.000^*^	

*P significant; ^a^ t test; ^b^ Mann–Whitney test; ^c^ Wilcoxon test

The preoperative V pattern exotropia patients’ deviation was 20 (min. 15, max. 45) PD for Group I and 20 (min. 15, max. 63) PD for Group II. The V pattern was collapsed with residual 2 (min. 0, max. 6) PD for Group I and 2 (min. 0, max. 10) PD for Group II, and there was a statistically significant difference between pre- and postoperative V pattern exotropia (*P* = 0.000) (Table 3).

**Fig. 3:**
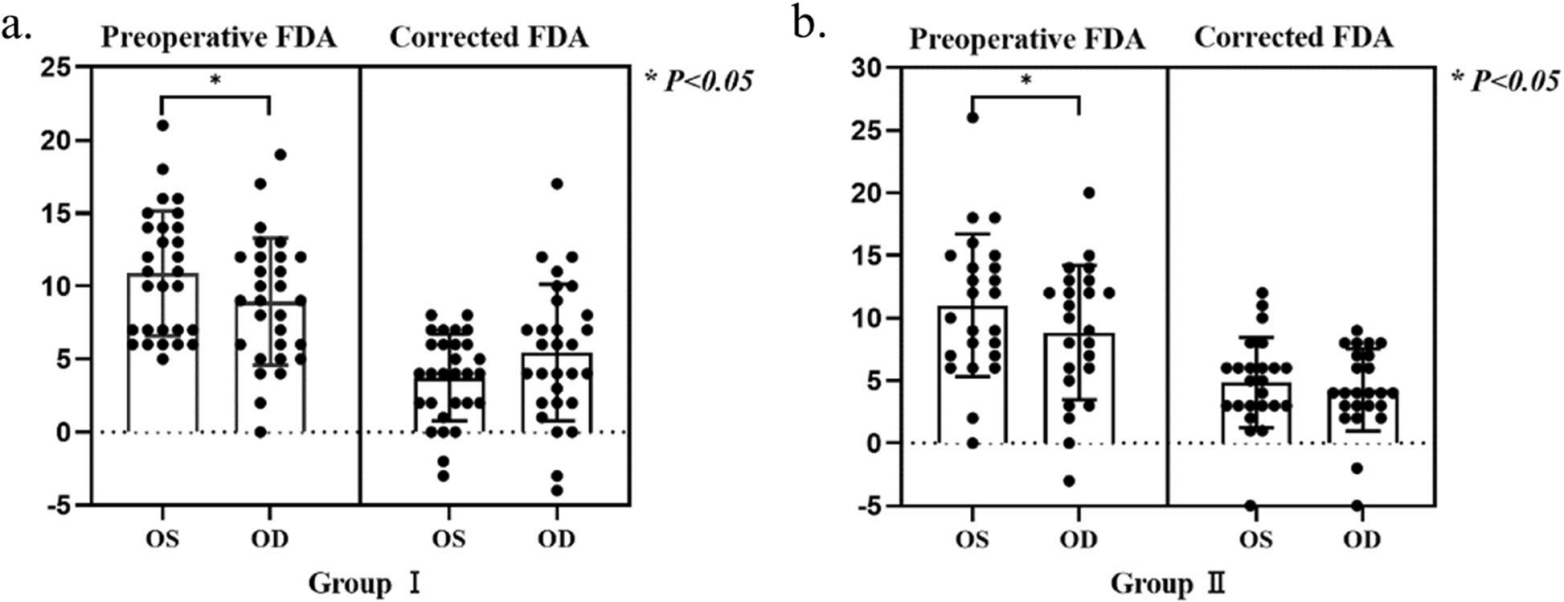
Results of preoperative and corrected FDA in V-pattern exotropia patients a. Comparison of preoperative and corrected FDA between the left eye and the right eye in Group I, b. Comparison of preoperative and corrected FDA between the left eye and the right eye in Group II. * Group I *p* = 0.029; Group II *p* = 0.038

Most patients included in this study had a degree of fundus extorsion. The preoperative unilateral FDA and surgically corrected FDA were also evaluated. The average preoperative FDA of the right eye and the left eye was (8.93 ± 4.34)° and (10.86 ± 4.27)°, respectively, in Group I. There was a significant difference in preoperative FDA between the right eye and the left eye in Group I (*P* = 0.029). The average preoperative FDA of the right eye and the left eye was (9.08 ± 4.92)° and (11.00 ± 5.69)° in Group II. A statistically significant difference in preoperative FDA between the right eye and the left eye in Group II was observed (*P* = 0.038). At the final follow-up, the mean amount of corrected FDA of the right eye and the left eye in surgery was (5.45 ± 4.68)° and (3.72 ± 2.96)° in Group I. No statistically significant difference in the amount of corrected FDA was detectable between the right eye and the left eye in Group I (*P* = 0.488). The amount of corrected FDA of the right eye and the left eye in surgery was (4.25 ± 3.27)° and (4.83 ± 3.61)° in Group II. No statistically significant difference was observed in the amount of corrected FDA between the right eye and the left eye in Group II (*P* = 0.579). Figure 3 shows changes in preoperative unilateral FDA and corrected FDA at the final follow-up postoperatively. Before the strabismus surgery, 10 of 53 subjects had stereopsis of ≤60 s of arc, 23 subjects had stereopsis of > 60 s of arc and 20 subjects had no stereopsis. Twenty-nine patients achieved improvement in stereopsis after the surgery.

## Discussion

At present, the exact etiologies of A and V patterns remain unknown. Many theories, including oblique muscle dysfunction, craniofacial anatomy abnormalities, innervation abnormalities and changes in the pulleys of rectus muscles, have been proposed to imply the occurrence of A and V patterns [2–6]. Oblique muscle dysfunction, proposed by Philip Knapp in 1959, is considered the most prominent and widely accepted theory [7]. Thus, IO weakening is the most effective procedure for V-pattern exotropia with IOOA. Many studies have evaluated the effects of different IO weakening operations on V-pattern exotropia. ErinG. Sieck et al. studied 357 eyes of patients with IOOA who underwent IO recession, anteriorization, and myotomy [18]. They found that 94.6%, 97.4%, 98.7% of eyes had alleviation in IOOA after recession, anteriorization, and myotomy of the IO. Finally, they concluded that the success rates of these three procedures were similar. Recession of the IO muscle can be adjusted according to the degree of IOOA. However, the recession procedure is complicated and requires a long operation time [19]. IO partial myectomy is a common and safe procedure for the treatment of IOOA. The procedure is simple and straightforward and requires a short operation time [20, 21].

Moreover, Manal Kasem et al. studied 40 primary IOOA of all grades of patients with esotropia or exotropia who underwent IO retroequatorial myopexy and myectomy [22]. They found that the collapse of the V-pattern was achieved in 69% and 57% patients in the myopexy and myectomy groups, respectively, with a statistically significant difference (*P* ≤ 0.001). They reported that two procedures were more effective in collapsing the V-pattern associated with IOOA than in eliminating IOOA. Attiat M Mostafa et al. proposed a randomized prospective study in 43 patients with unilateral IOOA who underwent unilateral or bilateral IO graded recession-anteriorization [23]. They reported that a successful outcome was achieved in 64.7% and 76.5% patients in the unilateral and bilateral IOOA groups, respectively (*p* = 0.452). They concluded that no significant difference was found between the two groups; however, bilateral surgery had a higher rate of causing IO undercorrection. E M Helveston reported that contralateral IOOA was aggravated postoperatively with unilateral IO weakening [9]. Manal Kasem et al. reported that 66.6% of patients with unilateral IOOA developed contralateral IOOA in the myectomy group (*P* < 0.001) [22]. Zhang W et al. performed IO myotomy or combined IO partial myectomy in 78 V pattern patients with IOOA, and only 9 eyes in 121 surgical eyes had residual grade 1 IOOA [24].

Currently, there is ongoing controversy about whether bilateral or unilateral surgery should be performed in patients with asymmetric IOOA. Moreover, there have been no studies exploring the effects of bilateral IO partial myectomy on V-pattern strabismus with bilateral symmetric IOOA and asymmetric IOOA. Our study evaluated the efficacy of bilateral IO partial myectomy in V-pattern exotropia patients with bilateral symmetric IOOA and asymmetric IOOA and compared it between the two groups.

Our results showed that IOOA was significantly improved in both groups before and after partial myectomy (*p* = 0.000 for Group I; *p* = 0.000 for Group II). There were 3 eyes in Group I and 2 eyes in Group II with residual grade 1 IOOA, and no IO underaction was observed. It is suggested that bilateral IO partial myectomy may be an effective and safe surgical approach for bilateral IOOA. The following conditions, including the presence of adhesion syndrome and a residual band of IO muscle, can lead to the occurrence of residual IOOA. In our study, small residual IO muscle bands were found during secondary surgical exploration. However, it is possible to prevent the above situations if the operation was gentle and inspected carefully after the IO muscles were hooked and the normal surrounding tissues were protected. In addition, the rate of collapse of the V pattern in Group I was similar to that in Group II. These results could be explained by the fact that IO partial myectomy caused marked weakening of the IO, especially in the field of elevation and adduction of the muscle.

As IO is the extortor of the globe, IOOA is often associated with globe extorsion. In this study, we found that the preoperative extorsion was not evenly distributed between the right eye and the left eye in the two groups, which was consistent with the research on 22 V pattern patients with IOOA by Yu X et al. [25].

In addition, the surgery corrected FDA was equally distributed between the right eye and the left eye in the two groups, indicating that there was an internal rotation tendency for FDA after binocular surgery compared with preoperative FDA. Noticeably, patients with IOOA may not have corresponding extorsion complaints, which can be explained by the appearance of compensatory head tilt and binocular fusion.

However, some limitations might also be mentioned. First, as it was a retrospective investigation, selection bias might have occurred. Second, a relatively small number of patients with V-pattern exotropia were included. A larger randomized and prospective study should be conducted in further research. Third, the duration of follow-up was short. To the best of our knowledge, this is the first study that has evaluated the efficacy of bilateral IO partial myectomy for V-pattern exotropia patients with IOOA and compared bilateral symmetric and asymmetric IOOA. The results can be of great significance in future investigations. The contrast between symmetric and asymmetric IOOA by the same procedure and the objective FDA indictor are two considerable strengths of this study. In addition, the combination of eye movement examination and quantitative evaluation of the ocular extorsion was used in this study to clarify the clinical diagnosis and evaluation.

## Conclusion

In conclusion, bilateral IO partial myectomy is an effective and safe method for V-pattern patients with symmetric and asymmetric IOOA, which can bring “symmetric” effectiveness in the reduction of FDA. It can potentially be used as a safe and successful treatment for V-pattern exotropia with bilateral IOOA. In addition, the FDA may be a promising index for evaluating fundus extorsion.

## Data Availability

The datasets used and/or analyzed during the current study are available from the corresponding author on reasonable request.

## Abbreviations

IOOA: Inferior oblique overaction
FDA: Forea-disc angle
IO: Inferior oblique
AES: Antielevation syndrome
SOOA: Superior oblique overaction

## References

[1] Kushner BJ (2010). Effect of ocular torsion on A and V patterns and apparent oblique muscle overaction. Arch Ophthalmol (Chicago, Ill: 1960).

[2] Kekunnaya R, Mendonca T, Sachdeva V (2015). Pattern strabismus and torsion needs special surgical attention. Eye (London, England).

[3] Dagi LR, MacKinnon S, Zurakowski D, Prabhu SP (2017). Rectus muscle excyclorotation and V-pattern strabismus: a quantitative appraisal of clinical relevance in syndromic craniosynostosis. Br J Ophthalmol.

[4] Lambert SR (2002). Are there more exotropes than esotropes in Hong Kong?. Br J Ophthalmol.

[5] Nowakowska O, Broniarczyk-Loba A, Loba PJ (2008). The reduction of A-V patterns with oblique muscles overaction in unilateral and bilateral surgery. Klin Ocz.

[6] Pallus A, Mustari M, Walton MMG (2019). Abnormal eye position signals in interstitial nucleus of cajal in monkeys with "a" pattern strabismus. Invest Ophthalmol Vis Sci.

[7] Knapp P (1959). Vertically incomitant horizontal strabismus: the so-called "a" and "V" syndromes. Trans Am Ophthalmol Soc.

[8] Costenbader FD, Kertesz E (1964). Relaxing procedures of the inferior oblique; a comparative study. Am J Ophthalmol.

[9] Ozsoy E, Gunduz A, Ozturk E (2019). Inferior oblique muscle overaction: clinical features and surgical management. J Ophthalmol.

[10] Awadein A, Gawdat G (2008). Bilateral inferior oblique Myectomy for asymmetric primary inferior oblique Overaction. J AAPOS..

[11] Guemes A, Wright KW (1998). Effect of graded anterior transposition of the inferior oblique muscle on versions and vertical deviation in primary position. J AAPOS.

[12] Clark RA, Miller JM, Demer JL (1998). Displacement of the medial rectus pulley in superior oblique palsy. Invest Ophthalmol Vis Sci.

[13] Lee YB, Rhiu S, Lee JY, Choi MY, Paik HJ, Lim KH, Choi DG (2017). Effect of horizontal rectus surgery for the correction of intermittent exotropia on sub-a or sub-V pattern. PLoS One.

[14] Urist MJ (1951). Horizontal squint with secondary vertical deviations. AMA Arch ophthalmology.

[15] Aghdam KA, Asadi R, Sanjari MS, Sadeghi A, Razavi M (2021). Comparing two inferior oblique weakening procedures: Disinsertion versus Myectomy. J Ophthalmic Vis Res.

[16] Chen X, Zhao KX, Guo X, Chen X (2008). Application of fundus photography in the diagnosis and curative effect evaluation of inferior oblique muscle overaction. Chin J Optometry Ophthalmol.

[17] Spierer A (1996). Measurement of cyclotorsion. Am J Ophthalmol.

[18] Sieck EG, Madabhushi A, Patnaik JL, Jung JL, Lynch AM, Singh JK (2020). Comparison of different surgical approaches to inferior oblique overaction. J Binocular Vision Ocular Motility.

[19] Lee DC, Lee SY (2017). Effect of modified graded recession and anteriorization on unilateral superior oblique palsy: a retrospective study. BMC Ophthalmol.

[20] Shipman T, Burke J (2003). Unilateral inferior oblique muscle myectomy and recession in the treatment of inferior oblique muscle overaction: a longitudinal study. Eye (Lond).

[21] Min BM, Park JH, Kim SY, Lee SB (1999). Comparison of inferior oblique muscle weakening by anterior transposition or myectomy: a prospective study of 20 cases. Br J Ophthalmol.

[22] Kasem M, Metwally H, El-Adawy IT, Abdelhameed AG (2020). Retro-equatorial inferior oblique myopexy for treatment of inferior oblique overaction. Graefes Arch Clin Exp Ophthalmol.

[23] Mostafa AM, Kassem RR (2018). Comparative study of unilateral versus bilateral inferior oblique recession/anteriorization in unilateral inferior oblique overaction. Eur J Ophthalmol.

[24] Zhang W, Zhao KX, Du CQ, Guo X (2002). Surgical treatment of V pattern exotropia. Chin J Pract Opht.

[25] Yu X, Mai G, Yu H, Deng D, Lin X, Chen J, Wu H (2003). Study on ocular torsion of V patterns strabismus. Eye science.

